# Impact of a Health Coach–Led, Text-Based Digital Behavior Change Intervention on Weight Loss and Psychological Well-Being in Patients Receiving a Procedureless Intragastric Balloon Program: Prospective Single-Arm Study

**DOI:** 10.2196/54723

**Published:** 2024-07-31

**Authors:** Paul M Sacher, Emily Fulton, Victoria Rogers, Julia Wilson, Marco Gramatica, Jennifer E Dent, Edo O Aarts, David Eccleston, Jan Willem Greve, Inge Palm-Meinders, Ram Chuttani

**Affiliations:** 1 Allurion Technologies Inc Natick, MA United States; 2 Allurion Kliniek Hilversum Netherlands; 3 MediZen Birmingham United Kingdom; 4 Nederlandse Obesitas Kliniek (Dutch Obesity Clinic) Huis Ter Heide Netherlands

**Keywords:** intragastric balloon, obesity, behavior change, health coaching, digital health, weight management, well-being, mobile phone

## Abstract

**Background:**

Digital health interventions show promise for weight management. However, few text-based behavior change interventions have been designed to support patients receiving intragastric balloons, and none have simultaneously evaluated weight loss, psychological well-being, and behavior change despite the crucial interplay of these factors in weight management.

**Objective:**

This study aims to assess whether a health coach–led, asynchronous, text-based digital behavior change coaching intervention (DBCCI) delivered to participants receiving an intragastric balloon and its aftercare program was feasible and acceptable to participants and supported improved outcomes, including weight loss, psychological well-being, and lifestyle behavior change conducive to weight loss maintenance.

**Methods:**

This 12-month, single-arm prospective study enrolled adults aged 21 to 65 years with BMI ≥27 kg/m^2^ receiving a procedureless intragastric balloon (PIGB) at 5 bariatric clinics in the United Kingdom and the Netherlands. Participants received the DBCCI and the clinic-led PIGB aftercare program (remotely delivered) for 6 months after PIGB placement and then no intervention for an additional 6 months. The DBCCI was an evidence-based, personalized intervention wherein health coaches supported participants via exchanged asynchronous in-app text-based messages. Over the 12-month study, we assessed percentage of total body weight loss and psychological well-being via self-administered validated questionnaires (Warwick-Edinburgh Mental Wellbeing Scale, Generalized Anxiety Disorder Scale, Impact of Weight on Quality of Life–Lite–Clinical Trials Version, Loss of Control Over Eating Scale–Brief, Weight Efficacy Lifestyle Questionnaire–Short Form, and Barriers to Being Active Quiz). Participant engagement with and acceptability of the intervention were assessed via self-reported surveys.

**Results:**

Overall, 107 participants (n=96, 89.7% female; mean baseline BMI 35.4, SD 5.4 kg/m^2^) were included in the analysis. Mean total body weight loss was 13.5% (SEM 2.3%) at the end of the DBCCI and 11.22% (SEM 2.3%) at the 12-month follow-up (*P*<.001). Improvements were observed for all psychological well-being measures throughout the 12 months except for the Generalized Anxiety Disorder Scale (improvement at month 1) and Barriers to Being Active Quiz (improvements at months 3 and 6). Surveys showed high levels of engagement with and acceptability of the DBCCI.

**Conclusions:**

This study provides evidence that the health coach–led, asynchronous, text-based DBCCI was engaging and acceptable to participants with overweight and obesity. The DBCCI, delivered alongside the PIGB and its aftercare program, supported improved weight loss outcomes and psychological well-being versus baseline and was associated with lifestyle behavior changes known to help achieve and maintain long-term weight loss and improved health outcomes. Follow-up findings suggest a potential need for longer-term, more intense coaching to focus on weight loss maintenance and support ongoing self-coaching. This could be achieved by leveraging generative artificial intelligence to provide ongoing automated behavior change coaching support to augment human-led care.

**Trial Registration:**

ClinicalTrials.gov NCT05884606; https://clinicaltrials.gov/study/NCT05884606

## Introduction

### Background

The effective management of obesity requires a multimodal approach based on evidence-based behavior change strategies to achieve lifestyle modification that are delivered alongside interventions including cognitive behavioral therapy (CBT) for obesity, medical nutritional therapy, bariatric interventions, and antiobesity medications, all on a personalized basis [1-5].

The psychological well-being of patients with obesity is crucial to the management of this disease. However, the role it plays is complex due to the bidirectional relationship between psychological correlates and weight outcomes [6]. How these psychological factors impact weight loss and subsequent weight maintenance remains unclear [7]. Compared with populations who are not living with obesity, many patients who perceive themselves as living with obesity show poorer psychological well-being [8]. In turn, these patients experience greater difficulties making and sustaining lasting behavior changes and tend to lose less weight than those with better psychological well-being [6,9]. Understanding the mechanisms through which psychological factors influence the formation of long-lasting lifestyle habits is imperative for the management of obesity [10].

Intragastric balloons are a nonsurgical treatment option for suitable patients with overweight and obesity. Similar to bariatric surgery and other medical options, it is recommended that treatment with intragastric balloons be delivered alongside lifestyle modification and behavioral support to achieve and maintain lifestyle changes conducive to sustained weight loss and improved health outcomes [11,12]. Among interventions to deliver lifestyle support and promote healthy lifestyle habits, digital health interventions have become increasingly attractive due to their advantages over face-to-face approaches. These include anytime access, anonymity, fewer interpersonal barriers related to social anxiety or weight-related stigma, affordability, and a reduction in health care costs by virtue of the potential for widespread scalability [13-15].

A growing number of studies have demonstrated the effectiveness of digital health coaching or digital behavior change interventions compared to in-person support in improving weight loss, behavior change, or psychological well-being outcomes in patients with overweight or obesity [16-19]. However, to the authors’ knowledge, no studies on digital behavior change interventions have assessed all 3 outcomes at the same time in this population. Given the complex interrelation between weight loss, behavior change, and psychological well-being, it would be relevant to consider and evaluate all 3 simultaneously. In addition, research on digital health interventions in patients eligible for metabolic or bariatric surgery—including those who receive intragastric balloons—is also lacking [15]. Furthermore, little is known about whether digital coaching interventions that are layered on top of a standard aftercare program and delivered entirely via text-based in-app messaging are acceptable to patients.

**Figure 1:**
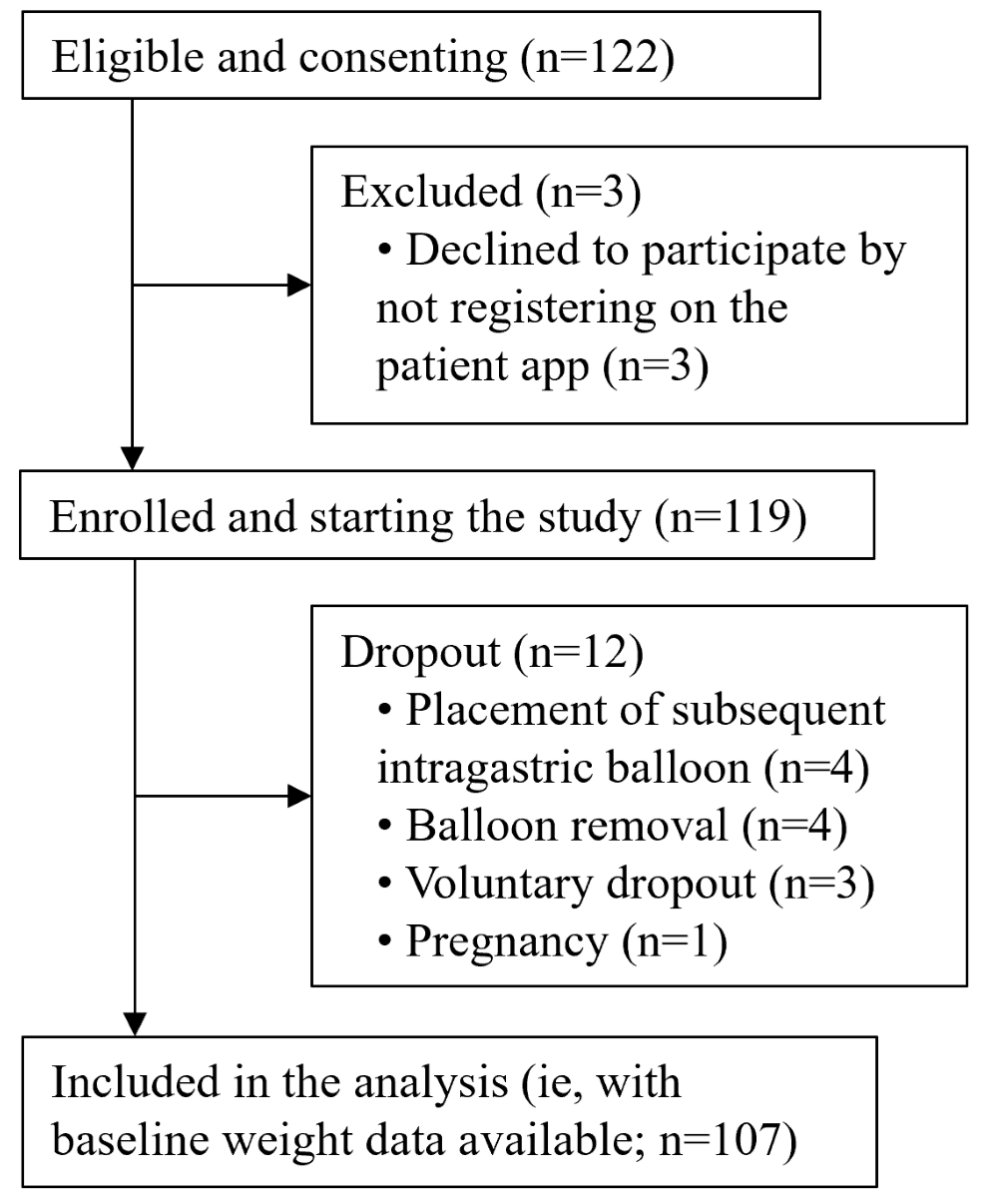
Participant flow diagram.

**Figure 2:**
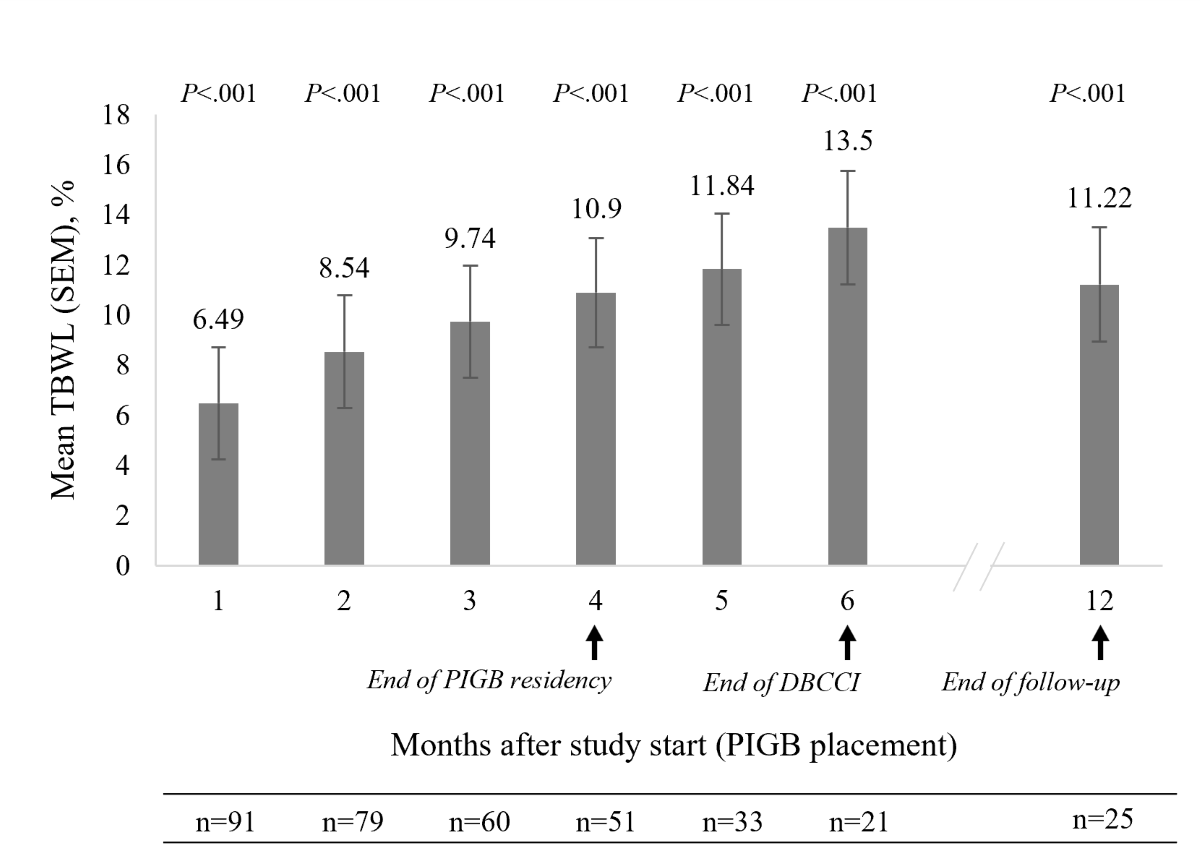
Total body weight loss (TBWL) over the study period. A multiple imputation method was used to handle missing weight data. TBWL was estimated using generalized estimation equation models adjusting for age, visit, and country. Error bars represent the SEM. P values are for the comparison versus baseline. DBCCI: digital behavior change coaching intervention; PIGB: procedureless intragastric balloon.

### Objectives

To provide evidence to address these gaps, this study aimed to assess a novel digital health coaching intervention in patients with overweight and obesity being treated with an intragastric balloon in combination with the balloon aftercare program (a clinic-led weight management program). In particular, this study aimed to assess whether (1) an asynchronous, text-based behavior change health coaching intervention delivered entirely remotely was feasible to implement and acceptable to this patient population; and (2) it was associated with improvements in weight loss, psychological well-being, and patient satisfaction, including factors related to behavior change.

## Methods

### Study Design

This was a single-arm, prospective study conducted in 3 bariatric clinics in the United Kingdom and 2 in the Netherlands between July 2021 and November 2022.

### Ethical Considerations

The ethics committee at the Central Committee on Research Involving Human Subjects in the Netherlands approved the study protocol (reference NL78284.096.21). The UK Health Research Authority confirmed that ethics committee approval was not required for UK sites as eligible participants were not National Health Service patients and no National Health Service sites or services were to be used. After reviewing the participant information sheet, all study participants provided written informed consent in person or via email before study enrollment on the day of procedureless intragastric balloon (PIGB) placement. Protective measures were taken to safeguard participant privacy, including anonymization and deidentification of participant data. Participants were not compensated for their participation in this study.

### Participants

Adults aged 21 to 65 years were eligible to participate in the study if they met all inclusion criteria: (1) providing informed consent; (2) having a BMI of ≥27 kg/m^2^; (3) weighing <180 kg; (4) having no contraindications for the use of the PIGB (Allurion Technologies; (Multimedia Appendix 1); (5) having received a PIGB in accordance with the approved indications for use on the day of potential enrollment; (6) owning an Android or iPhone smartphone; (7) being proficient in reading the English language; and (8) being willing to download the Allurion patient app, use the Allurion connected scale, and wear the Allurion smartwatch for the duration of the study. Study participants covered the cost for their treatment with the PIGB weight management program.

### Study Sites and Recruitment Process

A convenience sample of 5 bariatric clinics in the United Kingdom and the Netherlands was chosen. These included varied geographic locations and degrees of urbanization, clinic size, and number of balloon placements. The aim was to provide a representative sample of European participants to better generalize to the target population of patients receiving a PIGB. Eligible participants were given the participant information sheet in their local language and were invited to participate by clinic staff. Enrollment included downloading and registering on the patient app.

### Intervention

The digital behavior change coaching intervention (DBCCI) is a health coach–led behavior change intervention delivered via remote, asynchronous, in-app text-based messaging over 6 months. The DBCCI was delivered in combination with standard care—a clinic-led weight management program.

#### Description of the Clinic-Led Weight Management Program

Before study start, participants underwent an initial medical and dietary history and lifestyle assessment to determine eligibility for the intragastric balloon. At the baseline visit, participants received the PIGB, which is swallowed and requires no anesthesia or endoscopy for placement, making it possible to place in the outpatient setting [20-26]. Before and after filling it with 550 mL of distilled water, the correct positioning of the PIGB in the stomach is confirmed through x-ray visualization of its radiopaque components. In addition to the PIGB, which takes up space in the stomach and delays gastric emptying (to help participants feel fuller quicker on smaller portions and fuller for longer), the clinic-led weight management program also consists of remote patient monitoring and communication tools (smartphone app, wireless connected body composition scale, and smartwatch) to support patients during treatment and provide the clinic team with an opportunity to closely monitor their patients’ progress and intervene if needed. After approximately 4 months, the PIGB self-empties via a release valve that opens automatically, allowing the contents to empty into the stomach, and the collapsed balloon passes naturally through the gastrointestinal tract.

For 6 months after PIGB placement, participants received the PIGB aftercare program offered by the study clinics at time of study conduction. This program consisted of personalized symptom, nutritional, dietary, physical activity, and lifestyle management advice delivered by the clinic team, including a physician or a nurse (or both) and a registered dietitian or lifestyle coach. Between the baseline visit and month 6 after PIGB placement, participants had 4 to 6 telehealth consultations with their clinic team. Due to the COVID-19 pandemic, all provider-to-participant aftercare treatment and support were delivered remotely (via video or phone calls, email, and messaging apps such as WhatsApp [Meta Platforms]).

#### Description of the DBCCI

##### Overview

The DBCCI consisted of multicomponent, theoretically driven, evidence-based, and personalized behavior change coaching delivered remotely by health coaches via asynchronous one-to-one text-based messaging (it did not include telehealth interactions). The overall goal of the DBCCI was to facilitate behavior change to support weight management, including implementing behavioral actions related to diet, physical activity, sleep, self-regulation, and psychological well-being. As a personalized intervention, the aim of the DBCCI was to identify suitable behavioral actions that were realistic and achievable for each participant and apply behavior change techniques (BCTs) to support the participant in the process of successful habit formation. In particular, the DBCCI aimed to promote participant autonomy, enhance self-efficacy, and support the development of self-regulation skills to manage challenges and potential relapses to unhelpful patterns of behavior.

##### Development of the Intervention

The DBCCI was designed by the behavioral science team at Allurion Technologies using the behavior change wheel, an internationally recognized framework for behavior change intervention development [27]. As a first step, a literature review was conducted to identify appropriate and effective BCTs for weight management and common barriers to and facilitators of weight-related behavior change (the latter comprising psychological, social, environmental, physical, and cognitive aspects). This information was combined with previous quantitative and qualitative survey data collected from patients who had received the PIGB. All these data were then mapped onto the Capability, Opportunity, and Motivation–Behavior model (ie, a theoretical framework involving the 3 essential conditions that influence behavior change) and to the Theoretical Domains Framework (ie, the 14 domains within the Capability, Opportunity, and Motivation–Behavior model providing further details on the drivers of behavior) [27]. From this, relevant BCTs to help address the barriers to change and enable sustained behavior change were identified using the BCT Taxonomy version 1 [28]. These BCTs constituted the main components of the intervention, as described in the following section.

##### Components of the Intervention

Core BCTs were delivered to all participants and included goal setting, action planning, problem-solving, and self-monitoring (self-tracking) of behavior. Additional BCTs were offered on a personalized, ad hoc basis as required. Examples include restructuring the physical or social environment and providing prompts, cues, and rewards to action. To help identify suitable new behaviors (habits) to focus on, participants also received evidence-based “weight loss actions,” which were developed based on an adaptation of the weight loss actions described in the People Regulating Themselves to Achieve Weight Loss (PREVAIL) study [29].

The DBCCI provided participants with written guidance on the rationale for the weight loss actions and the core BCTs designed to help implement them. Whenever required, other techniques based on CBT and acceptance and commitment therapy [1,30] were also used by the health coaches, including support with cognitive restructuring, behavioral and emotional self-regulation, stress management, sleep hygiene, and relapse prevention. Motivational interviewing techniques [31] were applied where necessary to support participants expressing ambivalence about lifestyle change. In addition, whenever any specific balloon-related dietary or symptom management concerns arose or a request for personalized nutrition advice was made during the health coaching, the coaches referred participants back to their clinic teams.

##### Delivery of the Intervention and Access by Participants

The DBCCI started between the baseline and day 10 after PIGB placement and finished at the end of month 6 to coincide with the end of the clinic-led program (after which no further communication took place). As part of the DBCCI, participants were requested to download the patient app, weigh themselves using the connected scale at least weekly, wear the smartwatch as often as possible, and engage with their health coach as needed up to month 6.

The DBCCI was delivered by experienced health coaches trained in evidence-based BCTs who had an undergraduate or higher degree in health psychology, nutrition, physical activity, or a behavior change–related subject. Coaches had access to professional supervision from 2 psychologists trained in CBT and a senior registered dietitian throughout the DBCCI period. Of note, coaches did not use any of the psychological well-being outcome data collected during this study to personalize their coaching.

The DBCCI was delivered to participants via the patient app, whereas coaches and clinics used a separate web-based application designed for remote patient management, monitoring, and communication (Allurion Insights; Allurion Technologies).

Health coaches provided asynchronous personalized, text-based coaching support via messaging 7 days per week and responded to participant messages within 24 hours. Participants were closely monitored, and records were kept of the participants’ action plans and revisions, their goals, and any information relevant to their progress. These records were accessible to all health coaches. The frequency of support was participant led, with health coaches contacting participants at least once weekly to check in and provide support and feedback on their progress in relation to their weight change, physical activity, and sleep data collected as part of the PIGB aftercare support program. All participants were able to access the coaches daily for asynchronous support as needed during the 6-month period.

Participants received in-app notifications when a message was received. Health coaches were notified via the web-based application when messages were opened by a participant. Participants had access to the health coach messages and the electronic materials shared with them 7 days per week during the 6-month intervention period.

### Assessments and Outcomes

#### Weight

Weight data (in kilograms) were obtained via the connected scale at baseline (defined as time of first weight recording within 7 days from study start) and each month (defined as each 30-day period [–7 days to +7 days] after study start) except for month 12, in which weight data were obtained via the connected scale (11/25, 44% of the participants with available data) and via self-reporting through the participant-reported satisfaction surveys (14/25, 56% of the participants with available data).

#### Psychological Well-Being

The following self-administered validated questionnaires were used to assess the psychological well‑being of participants:

- The Warwick-Edinburgh Mental Wellbeing Scale (WEMWBS) is a 14-item internationally used measure of well-being in the general population [32,33]. Total scores range from 14 to 70, with higher scores indicating better well-being [33].
- The Generalized Anxiety Disorder Scale (GAD-7) is a 7-item scale widely used to measure anxiety in nonpsychiatric populations [34]. Total scores range from 0 to 21, with higher scores indicating more severe levels of anxiety associated with functional impairment [34].
- The Impact of Weight on Quality of Life–Lite–Clinical Trials Version (IWQOL-Lite-CT) scale is a 20-item measure of weight-related impact on physical, cognitive, and emotional functioning [35]. The IWQOL-Lite-CT includes 2 domains, for which scores are reported in this paper along with total score: the physical domain (7 items) and the psychosocial domain (13 items). IWQOL-Lite-CT total scores and scores for each domain range from 0 to 100, with higher values indicating higher levels of functioning. Score changes of ≥13.5 (physical domain), ≥16.2 (psychosocial domain), or ≥16.6 (total score) points indicate meaningful responses to treatment [36].
- The Loss of Control Over Eating Scale–Brief Version (LOCES-Brief) is a 7-item measure of perceived degree of control versus impulsivity for eating and overeating [37]. Total scores range from 0 to 28, with higher scores indicating less control over eating.
- The Weight Efficacy Lifestyle Questionnaire–Short Form (WEL-SF) is an 8-item measure of weight-related self-efficacy [38]. Total scores range from 0 to 80, with higher scores indicating greater confidence or self-efficacy.
- The Barriers to Being Active Quiz (BBAQ) is a 21-item measure of the cognitive, environmental, social, and health-related barriers to physical activity [39]. Total scores range from 0 to 63, with higher scores indicating a greater degree of difficulty in being active.

Questionnaires were completed via the Qualtrics software survey tool (Qualtrics International Inc) at baseline and at months 1, 3, 6, and 12. Invitations to complete the questionnaires were sent via patient app messaging and email.

#### Participant Engagement, Acceptability of the Intervention, and Impact on Behavior Change

To assess participant engagement and acceptability of the intervention, participant-reported satisfaction surveys were conducted to measure perceived usefulness of the intervention and its components and level of satisfaction with the intervention and their weight management journey overall. The surveys also included items to assess self-reported mediators of lifestyle behavior change, which are factors known to have a direct impact on successful behavior change (eg, having made a plan to change eating or physical activity habits). The surveys consisted of nonvalidated questions, with items rated on a 5-point Likert scale (0=“strongly disagree,” 1=“slightly disagree,” 2=“unsure,” 3=“slightly agree,” and 4=“strongly agree”) or an 11-point scale (0 to 10, with 0 as “not at all” and 10 as “completely”). The questions were developed with input from a multidisciplinary panel of experts in the field of obesity research and behavioral science. Participants completed the evaluation surveys through the Qualtrics survey tool at months 1, 3, 6, and 12; some items were not included at all time points depending on the intended measurement and its relevance and timing relative to the intervention. Invitations to complete the questionnaires were sent via patient app messaging and email.

### Statistical Analyses

Statistical analyses were conducted using R (version 4.1.1; R Foundation for Statistical Computing). All R packages used were included in the Comprehensive R Archive Network dated December 19, 2022 (latest version at the time of analysis). Imputation analyses were conducted using SAS (version 9.4; SAS Institute).

As this was a study for exploratory purposes, no formal study size or power calculations were performed a priori. On the basis of the average number of patients receiving the PIGB in the clinics that participated in the study, it was estimated that we would be able to recruit up to 150 participants during the recruitment period of the study.

Demographic characteristics (age, sex, ethnicity, body weight, BMI, BMI category, weight loss goal, and highest educational level) were summarized descriptively using frequency and proportion for categorical variables and mean and SD for continuous variables. BMI categories were defined as follows: 25 to <30 kg/m^2^ for overweight, 30 to <35 kg/m^2^ for obesity class I, 35 to <40 kg/m^2^ for obesity class II, and ≥40 kg/m^2^ for obesity class III [40].

The multiple imputation method was selected and applied to handle missing weight data. After data imputation, the least-square means for change in weight compared with baseline were obtained for each time point using a generalized estimation equation model that included weight as the dependent variable and visit, age, country, and visit-by-country interaction as covariates. Participant and intercept were treated as random effects. Least-square means for change in weight were then converted to an estimated mean percentage change (referred to as total body weight loss [TBWL] and expressed as a percentage). SEM TBWL was obtained using Taylor-based expansion, whereas 2-sided *P* values were derived from the generalized estimation equation model (with a .05 significance level). Weight loss maintenance was calculated as the percentage of mean TBWL at the end of the follow-up in relation to the mean TBWL at a given time point during the study.

Mean scores and SDs were calculated for each of the validated questionnaires to assess participants’ psychological well-being at each time point. Change in score over time was expressed as a difference score calculated as score at time point of interest minus score at baseline. Difference scores versus the baseline were tested using paired 2-tailed *t* tests with a .05 significance level. Mean difference scores, SDs, 95% CIs, and *P* values of the comparison versus baseline were obtained for all questionnaires and time points.

For the analysis of the participant-reported satisfaction surveys measuring feasibility, acceptability, and impact on behavior change, the number and percentage of respondents who scored items positively at months 1, 3, 6, and 12 were summarized. A positive score was defined as a score of ≥3 for question items rated on a 5-point (0-4) Likert scale and a score of ≥5 for items rated on an 11-point (0-10) Likert scale. An average of the percentage of positive responders during the DBCCI intervention (months 1, 3, and 6) was also calculated.

## Results

### Study Population

Of the 122 eligible participants who provided consent to take part in the study, 119 (97.5%) were enrolled and 107 (87.7%) were included in the analysis; reasons for dropout are detailed in Figure 1. The number of participants for whom data were available and included in the analyses of the outcomes at each time point is shown in Multimedia Appendix 2.

A total of 69.2% (74/107) of the participants received the intervention in the United Kingdom, and 30.8% (33/107) received the intervention in the Netherlands. Table 1 shows the demographic characteristics of the study population. Most participants (96/107, 89.7%) were female, with an average age of 41.8 (SD 10.6) years. The mean BMI was 35.4 (SD 5.4) kg/m^2^, and participants had an average TBWL goal of 20.8% (SD 5.6%) at baseline (Table 1).

**Table 1:** Demographic characteristics of the study population (N=107).

Characteristics		Values
Age (y), mean (SD)		41.8 (10.6)
Sex (female), n (%)		96 (89.7)
**Race and ethnicity, n (%)^a^**		
	Arab	1 (0.9)
	Asian	11 (10.3)
	Black	2 (1.9)
	Multiethnic	5 (4.7)
	White	64 (59.8)
	Other	24 (22.4)
Body weight (kg), mean (SD)		99.4 (18.6)
BMI (kg/m^2^), mean (SD)		35.4 (5.4)
**BMI category, n (%)^b^**		
	Overweight	12 (11.2)
	Obesity class I	46 (43)
	Obesity class II	32 (29.9)
	Obesity class III	17 (15.9)
Weight loss goal set by participant (percentage of baseline body weight), mean (SD)		20.8 (5.6)
**Highest educational level, n (%)^c^**		
	Secondary school	7 (6.5)
	Vocational education	10 (9.3)
	University degree	72 (67.3)
	Other	18 (16.8)

^a^“Arab” category includes Moroccan; “multiethnic” includes Antillean, mixed Netherlands, mixed United Kingdom, and Suriname; “White” includes Nederland and White; and “other” includes not available, not specified, and other.

^b^BMI categories were defined as 25 to <30 kg/m^2^ for overweight, 30 to <35 kg/m^2^ for obesity class I, 35 to <40 kg/m^2^ for obesity class II, and ≥40 kg/m^2^ for obesity class III [40].

^c^“University degree” includes college, bachelor’s degree, master’s degree, and PhD and “other” includes not available and other.

### Weight Loss

One month after study start, participants achieved a mean TBWL of 6.49% (SEM 2.2%) (Figure 2). The TBWL further increased to 10.9% (SEM 2.2%) at month 4 (when the PIGB residency ended) and peaked at 13.5% (SEM 2.3%) at month 6 (when both the clinic standard aftercare program and DBCCI ended). The TBWL remained at 11.22% (SEM 2.3%) at the 12-month follow-up—that is, 8 months after the end of the PIGB residency and 6 months after the end of the DBCCI (Figure 2). The TBWL was statistically significant versus baseline at all time points evaluated (*P*<.001). In terms of body weight loss maintenance, the mean TBWL at the 12-month follow-up represented 103% of the TBWL at month 4 (end of the PIGB residency) and 83.1% of the TBWL at month 6 (end of the DBCCI).

### Psychological Well-Being

#### WEMWBS Results

A mean WEMWBS score of 45.9 (SD 8.7) was observed at baseline (91/107, 85%; Table 2). During the intervention period, statistically significant increases in this score were observed at all time points, reaching a high of 51.2 (SD 7.9) at month 6, when the DBCCI ended (mean difference score vs baseline=4.7, 95% CI 1.8-7.6; *P*=.002). At the 12-month follow-up, mean scores slightly decreased to 48.0 (SD 10.7) but remained significantly higher than at baseline (*P*=.02).

**Table 2:** Psychological well-being outcomes over the study period (N=107).

Outcomes				Baseline		Time point after study start						
						Month 1		Month 3		Month 6 (end of DBCCI^a^)		Month 12 (end of follow-up)
**WEMWBS^b^**												
	Participants with available data, n (%)			91 (85)		69 (64.5)		58 (54.2)		42 (39.3)		43 (40.2)
	Absolute score, mean (SD)			45.9 (8.7)		49.5 (7.8)		50.6 (9.7)		51.2 (7.9)		48.0 (10.7)
	**Difference in score versus baseline^c^**											
		Participants with available data, n (%)	—^d^		68 (63.6)		57 (53.3)		41 (38.3)		39 (36.4)	
		Difference in score, mean (SD; 95% CI)	—		3.8 (7.0; 2.1 to 5.5)		4.0 (8.0; 1.9 to 6.1)		4.7 (9.3; 1.8 to 7.6)		3.7 (9.2; 0.7 to 6.7)	
		*P* value	—		*<.001* ^e^		*<.001*		*.002*		*.02*	
**GAD-7^f^**												
	Participants with available data, n (%)			90 (84.1)		69 (64.5)		58 (54.2)		42 (39.3)		43 (40.2)
	Absolute score, mean (SD)			6.0 (4.3)		4.8 (4.1)		5.3 (4.5)		5.1 (3.9)		5.4 (4.8)
	**Difference in score versus baseline^c^**											
		Participants with available data, n (%)	—		67 (62.6)		56 (52.3)		40 (37.4)		38 (35.5)	
		Difference in score, mean (SD; 95% CI)	—		−1.3 (3.9; −2.2 to −0.3)		−0.8 (4.2; −1.9 to 0.3)		−0.3 (4.6; −1.7 to 1.2)		−1.2 (4.9; −2.8 to 0.4)	
		*P* value	—		*.009*		.16		.73		.14	
**IWQOL-Lite-CT^g^**												
	Participants with available data, n (%)			91 (85)		69 (64.5)		58 (54.2)		41 (38.3)		42 (39.3)
	Absolute score, mean (SD)			45.2 (15.6)		56.5 (14.1)		62.8 (16.3)		63.4 (15.6)		57.5 (19.1)
	**Difference in score versus baseline^c^**											
		Participants with available data, n (%)	—		68 (63.6)		57 (53.3)		40 (37.4)		38 (35.5)	
		Difference in score, mean (SD; 95% CI)	—		11.8 (13.1; 8.6 to 14.9)		17.3 (14.9; 13.3 to 21.2)		19.2 (18.6; 13.3 to 25.2)		13.6 (17.0; 8.0 to 19.2)	
		*P* value	—		*<.001*		*<.001*		*<.001*		*<.001*	
**IWQOL-Lite-CT: physical domain**												
	Participants with available data, n (%)			91 (85)		69 (64.5)		58 (54.2)		41 (38.3)		42 (39.3)
	Absolute score, mean (SD)			55.9 (17.9)		65.7 (16.3)		73.5 (16.8)		72.5 (20.6)		67.8 (20.3)
	**Difference in score versus baseline^c^**											
		Participants with available data, n (%)	—		68 (63.6)		57 (53.3)		40 (37.4)		38 (35.5)	
		Difference in score, mean (SD; 95% CI)	—		10.8 (14.7; 7.3 to 14.4)		17.9 (14.8; 13.9 to 21.8)		16.9 (19.2; 10.7 to 23.0)		13.3 (17.7; 7.4 to 19.1)	
		*P* value	—		*<.001*		*<.001*		*<.001*		*<.001*	
**IWQOL-Lite-CT: psychosocial domain**												
	Participants with available data, n (%)			91 (85)		69 (64.5)		58 (54.2)		41 (38.3)		42 (39.3)
	Absolute score, mean (SD)			39.5 (17.7)		51.6 (16.3)		57.1 (19.0)		58.5 (16.0)		52.0 (21.0)
	**Difference inscore versus baseline^c^**											
		Participants with available data, n (%)	—		68 (63.6)		57 (53.3)		40 (37.4)		38 (35.5)	
		Difference in score, mean (SD; 95% CI)	—		12.3 (15.1; 8.6 to 16.0)		16.9 (17.6; 12.3 to 21.6)		20.5 (20.3; 14.0 to 27.0)		13.8 (17.8; 7.9 to 19.6)	
		*P* value	—		*<.001*		*<.001*		*<.001*		*<.001*	
**LOCES-Brief^h^**												
	Participants with available data, n (%)			91 (85)		69 (64.5)		58 (54.2)		41 (38.3)		41 (38.3)
	Absolute score, mean (SD)			25.0 (7.0)		13.9 (4.8)		16.6 (6.6)		16.5 (6.0)		18.1 (7.7)
	**Difference in score versus baseline^c^**											
		Participants with available data, n (%)	—		68 (63.6)		57 (53.3)		40 (37.4)		38 (35.5)	
		Difference in score, mean (SD; 95% CI)	—		−10.8 (8.0; −12.8 to −8.9)		−8.3 (8.6; −10.5 to −6.0)		−8.2 (7.4; −10.6 to −5.8)		−6.3 (6.7; −8.5 to −4.1)	
		*P* value	—		*<.001*		*<.001*		*<.001*		*<.001*	
**WEL-SF** ^i^												
	Participants with available data, n (%)			83 (77.6)		68 (63.6)		57 (53.3)		41 (38.3)		40 (37.4)
	Absolute score, mean (SD)			40.8 (15.6)		51.1 (16.4)		50.7 (17.0)		53.4 (13.4)		47.0 (19.5)
	**Difference in score versus baseline^c^**											
		Participants with available data, n (%)	—		62 (57.9)		52 (48.6)		37 (34.6)		33 (30.8)	
		Difference in score, mean (SD; 95% CI)	—		11.3 (15.4; 7.4 to 15.2)		11.3 (18.1; 6.3 to 16.3)		9.2 (17.6; 3.4 to 15.1)		6.9 (16.3; 1.1 to 12.6)	
		*P* value	—		*<.001*		*<.001*		*.003*		*.02*	
**BBAQ^j^**												
	Participants with available data, n (%)			90 (84.1)		66 (61.7)		57 (53.3)		40 (37.4)		40 (37.4)
	Absolute score, mean (SD)			22.9 (10.9)		20.6 (10.5)		17.4 (11.8)		14.3 (9.8)		21.8 (14.2)
	**Difference in score versus baseline^c^**											
		Participants with available data, n (%)	—		65 (60.7)		56 (52.3)		39 (36.4)		36 (33.6)	
		Difference in score, mean (SD; 95% CI)	—		−1.7 (8.0; −3.7 to 0.3)		−4.1 (11.0; −7.0 to −1.2)		−5.6 (7.7; −8.1 to −3.1)		−1.2 (9.2; −4.3 to 2.0)	
		*P* value	—		.09		*.007*		*<.001*		.45	

^a^DBCCI: digital behavior change coaching intervention.

^b^WEMWBS: Warwick-Edinburgh Mental Wellbeing Scale.

^c^Difference scores were calculated as score at the time point of interest minus score at baseline.

^d^Not applicable.

^e^Italicization denotes statistically significant difference versus baseline (*P*<.05).

^f^GAD-7: 7-item Generalized Anxiety Disorder Scale.

^g^IWQOL-Lite-CT: Impact of Weight on Quality of Life–Lite–Clinical Trials Version.

^h^LOCES-Brief: Loss of Control Over Eating Scale–Brief Version.

^i^WEL-SF: Weight Efficacy Lifestyle Questionnaire–Short Form.

^j^BBAQ: Barriers to Being Active Quiz.

#### GAD-7 Results

The mean GAD-7 score at baseline was 6.0 (SD 4.3; 90/107, 84.1%; Table 2), which significantly decreased to 4.8 (SD 4.1) at month 1 (mean difference=−1.3, 95% CI −2.2 to −0.3; *P*=.009). From month 3, mean scores ranged between 5.1 (SD 3.9) and 5.4 (SD 4.8), with no significant differences compared with baseline (Table 2).

#### IWQOL-Lite-CT Results

The total IWQOL-Lite-CT mean score at baseline was 45.2 (SD 15.6; 91/107, 85%; Table 2). Scores gradually and significantly increased up to 63.4 (SD 15.6) at month 6, when the DBCCI ended (mean difference=19.2, 95% CI 13.3-25.2; *P*<.001). At the 12-month follow-up, the mean score decreased to 57.5 (SD 19.1) but remained significantly higher than at baseline (*P*<.001).

Scores for the physical and psychosocial domains of the IWQOL-Lite-CT scale also increased during the study. At baseline, the mean IWQOL-Lite-CT physical domain score was 55.9 (SD 17.9; 91/107, 85%; Table 2), which significantly increased during the DBCCI intervention (mean difference at month 6=16.9, 95% CI 10.7-23.0; *P*<.001). At month 12, mean scores modestly decreased to 67.8 (SD 20.3) but remained significantly higher than at baseline (*P*<.001). A similar change over time was observed for the IWQOL-Lite-CT psychosocial domain, with a baseline mean score of 39.5 (SD 17.7; 91/107, 85%; Table 2) that increased progressively until month 6 (mean difference=20.5, 95% CI 14.0-27.0; *P*<.001). Mean scores slightly decreased to 52.0 (SD 21.0) at the 12-month follow-up; however, mean scores at all time points were significantly higher than at baseline (*P*<.001).

#### LOCES-Brief Results

The mean LOCES-Brief score at baseline was 25.0 (SD 7.0; 91/107, 85%; Table 2). This decreased significantly to 13.9 (SD 4.8) at month 1 (mean difference=−10.8, 95% CI −12.8 to −8.9; *P*<.001) and then remained stable at 16.5 (SD 6.6) to 16.6 (SD 6.0) up to month 6, with statistically significant reductions at all time points compared with baseline (*P*<.001). At month 12, the mean absolute score increased to 18.1 (SD 7.7) but remained significantly lower than at baseline (*P*<.001).

#### WEL-SF Results

The mean score for the WEL-SF at baseline was 40.8 (SD 15.6; 83/107, 77.6%; Table 2). Mean scores increased to 53.4 (SD 13.4) at month 6 (mean difference=9.2, 95% CI 3.4-15.1; *P*=.003) and then decreased to 47.0 (SD 19.5) at month 12. WEL-SF scores remained significantly higher at all time points compared with baseline (*P*<.001 at months 1 and 3, *P*=.003 at month 6, and *P*=.02 at month 12; Table 2).

#### BBAQ Results

The mean BBAQ score at baseline was 22.9 (SD 10.9; 90/107, 84.1%; Table 2). After a modest, non–statistically significant decrease at month 1, mean scores significantly and steadily decreased until month 6, when they reached a minimum of 14.3 (SD 9.8; mean difference=−5.6, 95% CI −8.1 to −3.1; *P*<.001). At month 12, mean scores increased to 21.8 (SD 14.2), with no statistically significant difference compared to baseline (Table 2).

### Participant Engagement, Acceptability of the Intervention, and Impact on Behavior Change

The detailed results on participant engagement, acceptability, and impact on behavior change are presented in tabular format in Multimedia Appendix 3. In general, participants reported high levels of satisfaction with the DBCCI. On average, 81.9% gave a positive score (ie, agreed, be it slightly or strongly) to the following item: “I have found the health coaching useful/helpful.” Such percentage of agreement remained high until month 6, when the DBCCI ended.

An average of 81.6% of participants agreed with the following items: “I feel supported by my health coach towards meeting my weight goals” and “My study health coaches helped me to meet my weight goals.” Percentages were higher at month 1 (54/63, 86%) than at month 6 (30/39, 77%). Throughout months 1 to 6, an average of 73% of participants agreed with the following item—“My health coach has helped me develop strategies to lose weight”—with percentages increasing from 69% (43/62) at month 1 to 80% (32/40) at month 6.

A total of 68% (44/65) of participants at month 1 and 66% (37/56) at month 3 agreed with the following item: “I feel confident that I can reach my goal weight and maintain it.” In relation to that, 91% (60/66) of participants at month 1 and 91% (52/57) at month 3 agreed with the following item: “I feel confident about making changes to help me lose weight.” When asked at months 6 and 12, the percentages of participants who agreed with the following item—“I feel confident about maintaining my weight loss”—dropped to 58% (23/40) and 45% (18/40), respectively.

An average of 65.4% of participants agreed with the following item—“I have been able to put the information and actions from the articles (weight loss actions) into practice”—with higher rates at earlier than at later time points (49/64, 77%; 34/54, 63%; and 21/37, 57% at months 1, 3, and 6, respectively). In terms of mediators of behavior change, 85% (34/40) of participants or more agreed with the following item—“I have made a plan to change my eating habits”—and 82% (45/55) or more agreed with the following item—“I have made a plan to change my physical activity habits”—when asked during the DBCCI. Rates dropped to 78% (31/40) and 65% (26/40), respectively, at month 12.

When asked about barriers to reaching a goal weight, 84.4% of participants on average (months 1-6) agreed with the following item: “I am aware of some of the barriers to me reaching my weight goals.” This percentage was high at months 1 (59/66, 89%) and 3 (51/57, 89%) and then decreased to 55% (22/40) at month 12. On average, during the 6-month DBCCI, 66.2% of participants agreed with the following item—“My health coaching was personalized to my needs”—with the percentage being highest at months 1 (45/63, 71%) and 3 (41/56, 73%) and then decreasing to 54% (21/39) at month 6.

When asked the following—“How satisfied have you been tracking your weight, using the connected scales, on a scale from 0 (not at all satisfied) to 10 (completely satisfied)?”—an average of 85.4% of participants over the 6-month DBCCI answered positively, whereas 96% (23/24) did so at month 12.

## Discussion

### Principal Findings

This study provides evidence that the health coach–led DBCCI, delivered entirely remotely via text-based messaging, was feasible to implement and was acceptable to participants with overweight or obesity who took part in a clinic-led weight management program. The DBCCI, along with the PIGB aftercare program, was associated with improved weight loss and sustained psychological well-being outcomes over the 12-month study follow-up period compared with baseline. In addition, participants reported high levels of engagement with and acceptability of the health coaching provided. Many reported having made behavior change plans, feeling more confident about lifestyle change, and having put what they had learned into practice.

### Weight Loss

The mean 10.9% of TBWL at the end of the PIGB residency (month 4) in this study falls in the range of 10% to 15% mean TBWL at balloon passage reported in other PIGB studies [20,21,23,41-43]. The mean values of TBWL being higher in other PIGB studies could be explained by more intense support programs in those studies compared to this one. For instance, in the registry-based study by Ienca et al [20], which reported a 14.2% TBWL at month 4, all participants were reportedly recruited from clinics that provided their standard aftercare program with more intense and frequent support to patients compared to that provided in this study. A similar situation applies to a prospective, BMI-matched controlled study by Raftopoulos et al [43], which combined the PIGB with a high-intensity, 12-month aftercare program and reported a 14.9% TBWL at month 4. Hence, more intense support to participants appears to lead to higher values of TBWL at balloon passage.

After the end of the PIGB residency, participants in this study continued losing weight steadily for 2 months until the end of the DBCCI (mean TBWL at month 6=13.5%). One could argue that this continuous weight loss between months 4 and 6 may have been attributed (to some degree) to the DBCCI, although no direct causality can be assumed as this study did not include a control group. Among the previous PIGB studies that assessed TBWL from the end of the PIGB residency to month 6, Jamal et al [21] reported a change from 10.7% to 10.9% in a noncontrolled, single-center study, whereas the aforementioned study by Raftopoulos et al [43] reported a change from 14.9% to 15.3%.

Regarding long-term weight loss outcomes, previous PIGB studies have reported TBWL from month 4 (end of PIGB residency) to month 16 (ie, 12 months after the end of the PIGB residency) with varied results: from 10.7% to 7.9% in a single-center study [21]; from 13.8% to 10.1% in a noncontrolled multicenter study [44]; and from 13.9% to 13.4% in a noncontrolled, international multicenter study [45]. In our study, the TBWL at month 12 (longest follow-up time point) was 11.22%, which represents a sustained weight loss compared with the 10.9% TBWL observed at the end of the PIGB residency (weight loss maintained at month 12 vs month 4=103%). The aforementioned PIGB study by Raftopoulos et al [43] reported a TBWL improvement from 14.9% at month 4 to 16.9% at month 12, which represents a 113% weight loss maintenance at month 12 versus month 4. These results of the study by Raftopoulos et al [43] at month 12 could possibly be due to the combination of the PIGB with a high-intensity aftercare program delivered until month 12 [43], which is in line with the idea that more intense and continued support to patients beyond PIGB residency seems to provide more sustained impact in the long term (see the “Implications for Practice” section).

### Psychological Well-Being

A statistically significant impact of the DBCCI combined with the PIGB aftercare program was observed on several measures of psychological well-being. In particular, we found improved well-being, mood, weight-related quality of life, and increased self-efficacy and control over eating throughout the study. A small reversion in scores across most of these measures was detected between months 6 and 12; however, this is to be expected given that the coaching support ended at month 6. Despite this, most scores at month 12 remained significantly improved compared to baseline, indicating a lasting impact of the intervention. Previous studies have suggested that impaired psychological well-being is associated with weight regain and may hinder healthy behavior promotion among patients with obesity [9]. Therefore, it could be argued that the positive outcomes observed in this study are likely to help contribute to lasting weight loss by facilitating a greater actioning of behavior change strategies in the long term, improved self-regulation, and reduced emotional eating.

Well-being scores at baseline (based on the WEMWBS) were slightly below values reported in the general population (45.9 vs 51.0 [33]), as might be expected in people with overweight or obesity seeking treatment. To the authors’ knowledge, there is a paucity of studies measuring psychological well-being using the WEMWBS in people living with obesity specifically; therefore, direct comparisons with a similar cohort are not possible. In this study, WEMWBS scores improved during the DBCCI, reaching values comparable to those in general population at months 3 and 6.

Baseline GAD-7 scores in this study were slightly higher than those reported before surgery in a similar population of patients undergoing bariatric surgery published elsewhere (6.0 compared to 5.6 [46]). Nonetheless, both studies report only mild anxiety levels according to established cutoff points for the GAD-7 [34]. Anxiety levels in this study significantly decreased at month 1 versus baseline. This could reflect a positive effect of the DBCCI combined with the PIGB aftercare program or else a relief at the successful balloon placement and cessation of initial symptoms that could follow. Further research is needed to fully interpret this observation. As of month 3, no further significant changes versus baseline were noted in GAD-7 scores.

In terms of weight-related quality of life, IWQOL-Lite-CT scores at baseline were lower (worse) than in a comparative study of a similar population (45.2 vs 63.49) [36]. It is unclear why we see this difference, and further research is needed to explore this. Nevertheless, changes in IWQOL-Lite-CT scores (difference score) at months 3 and 6 were above the defined threshold for meaningful response to treatment for all 3 scores—total, physical, and psychosocial—and higher than those observed in other studies [36]. This suggests that the DBCCI and PIGB aftercare program produced a meaningful improvement in quality of life. However, 6 months after the end of the DBCCI, although significantly improved compared to baseline, the changes in score were no longer clinically meaningful based on published clinical thresholds [36], emphasizing again that more research is needed to determine how improvements in quality of life are better maintained.

Weight-related self-efficacy—which includes participants’ self-reported belief in their ability to put behavioral actions into practice—significantly improved at all time points over the course of the study. The highest improvement in WEL-SF scores was observed toward the end of the DBCCI (month 6). At month 12, scores decreased but remained significantly higher than at baseline, indicating a lasting effect of the intervention well after it finished. Given that self-efficacy is thought to play a crucial role in effective behavior change [47-49], it could be argued that the DBCCI combined with the PIGB aftercare program may contribute to successful behavior change. When comparing with WEL-SF scores obtained in a sample of patients living with obesity being considered for bariatric surgery [38], participants in this study presented with lower (worse) self-efficacy at baseline (average WEL-SF score of 40.8 vs 54.3 [38]).

The decrease in LOCES-Brief scores at all time points during this study indicates that the DBCCI combined with the PIGB aftercare program may have increased the level of control that participants felt they had over their eating. Unfortunately, no LOCES-Brief data have been published in patients from comparable populations, which precludes comparison of our results.

BBAQ scores (barriers to physical activity) at months 3 and 6 were lower than at baseline, indicating fewer difficulties related to being active and a potential positive effect of the DBCCI and PIGB aftercare program. At month 12, scores returned to near-baseline levels, which suggests the need to better target barriers to sustained physical activity behavior change specifically. No BBAQ data have been published in a comparable population, so these results cannot be compared with those of other studies.

### Impact on Behavior Change

The participant satisfaction surveys included items that allowed us to assess mediators of lifestyle behavior change. For example, most participants reported making an action plan to adopt new eating and physical activity habits within the first months of the intervention. This is a positive outcome given that this BCT has been proven to significantly increase the likelihood of sustained behavior change [50]. In addition, at least 89% (59/66) of participants reported a good understanding of their personal barriers to reaching their weight goals early in the study; however, this understanding worsened over time. Participants may have discovered that the presumed barriers were not impeding behavior change as initially thought in practice. This emphasizes the importance of coaching models that use behavioral science strategies, supporting patients in uncovering the often subconscious drivers and barriers to health behavior change [51].

Most of the participants in this study reported feeling supported or helped by their health coach and confident about making changes to their lifestyle behaviors, although slight drops were observed at month 6, which could be expected with the end of the intervention approaching. Participants might have feared a return to old habits or a weight plateau or regain. Guidance on how to identify other sources of social support after the intervention and how to continue their own “self-coaching” practice using the techniques learned could be given greater emphasis in a future iteration of the DBCCI.

### Implications for Practice

Even though the TBWL observed in this study is in line with results of other PIGB studies [20,23,41-43], the desired weight loss goal of participants at baseline (20.8% of body weight on average) suggests that participants’ goals may not have been in line with what studies suggest is realistic and what medical associations recommend in their official guidelines [52,53]. Therefore, participants may have set unrealistic and unattainable goals in this study, which could have led to disappointment. Patients’ unrealistic expectations and unhelpful beliefs about what they “should” achieve may benefit from cognitive therapeutic approaches, which support participants in developing a mindset that is self-compassionate; balanced; and more realistic about the need for a trial-and-error, small-steps approach to behavior change to achieve lifelong change and weight management success [1,54].

Following up with participants at 12 months allowed us to assess the extent to which outcomes changed after the end of the DBCCI. In terms of weight outcomes, the TBWL at the end of follow-up was statistically significant compared with baseline and similar to the TBWL at the end of the PIGB residency (weight loss maintained vs month 4=103%); however, TBWL at the end of follow-up was lower than at the end of the DBCCI (weight loss maintained vs month 6=83.1%). This observation, together with the results obtained in terms of participant engagement and behavior change and its impact, reflects the common trajectory for habit change—success in the early stages when participant motivation is high followed by challenges to sustaining this in the long term [55]. These data suggest a need for longer coaching support, greater focus on weight maintenance, and “self-coaching” promotion and practice. CBT techniques, incorporated specifically at the latter stages of support, can be introduced to help maintain self-regulatory behaviors associated with weight loss maintenance [1]. Our findings highlight the need for and importance of this intensive support at balloon passing and DBCCI end, when weight maintenance becomes paramount.

### Remote Aspect of the Intervention and Future Directions

Aside from the balloon placement, both the DBCCI and clinic-led PIGB aftercare program were delivered entirely remotely. The TBWL outcomes in this study were comparable to those obtained in other PIGB studies with in-person PIGB aftercare program support [23,41,42], and the remote intervention was associated with positive outcomes and high levels of acceptability and participant satisfaction. This suggests that remote delivery can be as effective as in-person patient support, which is in line with literature in the field comparing these approaches. A study reported similar effectiveness of web-based versus traditional face-to-face aftercare support programs following placement of an intragastric balloon [56]. Bus et al [16] found no significant weight differences of in-person versus web-based health coaching. Furthermore, a systematic review found no differences in weight or BMI changes between web-based and offline interventions for weight loss and lifestyle habit changes in adults living with overweight and obesity [57].

Although a number of evidence-based BCTs were included in the DBCCI, their effectiveness was dependent on participant engagement with coaching messages delivered asynchronously, which may have affected their accessibility and impact. Furthermore, the DBCCI (and all patient communication) ended at month 6 after PIGB placement. Had there been continued support up to 12 months, engagement until the end of the study is likely to have been higher. Providing this support in an instant, synchronous manner would likely improve accessibility and patient engagement even further.

There is early evidence that generative artificial intelligence (AI) conversational agents can deliver automated, immediate, text-based behavior change interventions and positively impact lifestyle behaviors, including in populations living with overweight and obesity [58,59]. If proven safe and effective in this application, AI-driven automated coaching support could be developed and implemented in a remote behavior change coaching intervention for weight management. AI also provides opportunities for scaling weight management interventions across cultures and different languages [58,59], and it opens up the possibility of augmenting human-led care to reduce the demand on humans—for example, implementing automated health coaching check-ins between in-person consultations. Future research on the use of AI-driven technologies to automate patient care, including delivery of behavior change interventions for weight management, is needed within well-designed clinical trials. Importantly, as with any new technologies, ensuring safety and efficacy is crucial.

### Limitations and Strengths of This Study

This study has some limitations. Its exploratory nature and single-arm design mean that no causality between intervention and outcomes can be determined. In addition, the combined intervention (DBCCI plus PIGB aftercare program) adds an extra layer of difficulty in attributing causality to one component or another or their combination (eg, as part of their standard PIGB aftercare program, clinic teams were likely to use certain BCTs while providing lifestyle advice, yet these BCT components were not itemized, and their potential impact on outcomes, if any, is difficult to assess). However, using a combined, multimodal intervention such as the one in this study is the approach that best aligns with real-world practice and weight management recommendations [2-5]. As mentioned previously, there seem to be greater weight loss mean values in other PIGB studies where a more intense and frequent PIGB aftercare program was delivered [20,43] compared with the aftercare program provided by the participating clinics at the time of this study (during the COVID-19 pandemic). On the basis of this and other scientific evidence collected, current best practices for the PIGB aftercare program involve high-quality and higher-intensity programs. This is to be considered when interpreting this study with other PIGB studies or with results obtained with this aftercare program in the clinical practice setting.

The number of participants who provided weight data at later time points of this study decreased over time (eg, 25/107, 23.4% at month 12 vs 107/107, 100% at baseline). This is in line with what has been reported in other studies of remotely delivered interventions [60-63], in follow-up appointments after bariatric surgery [64], or in trials of treatments for managing obesity, where up to 85% attrition after 12 months has been reported [65]. To account for missing weight data in this study, we used an imputation method at all time points. Nonetheless, we acknowledge that the smaller number of participants with available weight data at month 12 may not represent the whole cohort. Since this study was conducted, to help improve patient adherence to the weight management program, an AI model has been implemented to highlight to clinic teams which of their patients are not predicted to achieve minimally sufficient weight loss so that the clinic team can intervene early in the program with those patients [66]. In addition, weighing reminder notifications have been introduced in the patient app of the weight management program to encourage higher rates of self-monitoring of weight. In any case, despite the lower amount of weight data available at month 12 in this study, a greater proportion of participants were still engaged with the study at the end of follow-up, as shown by the higher number of participants (compared with weight data) who did complete the psychological well-being questionnaires and nonvalidated surveys at month 12 (43/107, 40.2% and 40/107, 37.4%, respectively).

Another limitation of this study is the fact that, at the time of study conduction, it was not possible to collect accurate data on behavior change self-monitoring (eg, tracking). Future research would benefit from assessing this outcome. In terms of methodology, some items used to measure feasibility, participant engagement, and impact on behavior change, which were rated via a Likert scale, may be difficult to interpret given the repeated-measure testing used. For instance, making a plan for eating and physical activity is a dichotomous variable—one either makes or does not make a plan. Participants may have made a plan at time points earlier than month 12, thus scoring low at month 12 (when no new plans were needed). In addition, this study may have suffered from selection bias—the fact that participants recruited in this study paid for their PIGB treatment might have made them more committed to the treatment than those undergoing reimbursable bariatric procedures. In any case, this circumstance is representative of the typical patient population receiving a PIGB in the United Kingdom and the Netherlands at the time. However, related to this, one should apply caution when generalizing the study findings to other populations worldwide given the demographic characteristics of the study population (eg, most participants were of White ethnicity). Such limited external validity is due to the exploratory nature of this study; future research in larger, more diverse populations would help address this limitation.

The main strength of this study is the obtention of data under real-world circumstances, in clinical practice and in a representative sample of patients undergoing PIGB treatment in the study countries. In addition, this study reports exhaustive data on psychological well-being outcomes using instruments for which little published data exist in this specific population, which contributes new evidence to the field and a pool of data for future comparison in similar populations. Finally, as discussed previously, the use of a text-based approach in this study lays down the potential for automation and application of AI-driven technologies to this type of intervention.

### Conclusions

This study provides evidence that a health coach–led digital behavioral intervention delivered via asynchronous text-based messaging was feasible, acceptable, and satisfactory to participants and appears to support improved weight loss, psychological well-being, and mediators of lifestyle behavior change that could be conducive to weight loss and maintenance. Insights obtained during this study, along with the text-based nature of the remote intervention described in this paper, open the door to the possibility of developing behavior change coaching approaches that take advantage of AI-driven automated support. Future research is needed to evaluate the benefits, risks, and impact of such approaches in supporting patients living with overweight and obesity.

## Appendix

Multimedia Appendix 1 Contraindications for the use of the procedureless intragastric balloon system.

Multimedia Appendix 2 Attrition diagram.

Multimedia Appendix 3 Percentage of participants who scored positively each item of the participant-reported satisfaction surveys to measure engagement, acceptability of the intervention, and impact on behavior change.

## Abbreviations

AI: artificial intelligence
BBAQ: Barriers to Being Active Quiz
BCT: behavior change technique
CBT: cognitive behavioral therapy
DBCCI: digital behavior change coaching intervention
GAD-7: Generalized Anxiety Disorder Scale
IWQOL-Lite-CT: Impact of Weight on Quality of Life–Lite–Clinical Trials Version
LOCES-Brief: Loss of Control Over Eating Scale–Brief Version
PIGB: procedureless intragastric balloon
PREVAIL: People Regulating Themselves to Achieve Weight Loss
TBWL: total body weight loss
WEL-SF: Weight Efficacy Lifestyle Questionnaire–Short Form
WEMWBS: Warwick-Edinburgh Mental Wellbeing Scale
