# Supplementary material for: Impact of a Health Coach–Led, Text-Based Digital Behavior Change Intervention on Weight Loss and Psychological Well-Being in Patients Receiving a Procedureless Intragastric Balloon Program: Prospective Single-Arm Study
Source: JMIR Form Res. 2024 Jul 31;8:e54723. doi: 10.2196/54723 (PMC11325120; doi:10.2196/54723)
Supplement: Multimedia Appendix 1 [file formative_v8i1e54723_app1.pdf]

Multimedia Appendix 1: Contraindications for the use of the procedureless intragastric balloon system.

Contraindications [1]

Difficulty swallowing (dysphagia):

- Any abnormal swallowing mechanism from an esophageal motility disorder such as achalasia, scleroderma, or diffuse esophageal spasm
- History of any structural esophageal abnormality such as a web, stricture, diverticulum, or para esophageal hernia

Conditions that predispose to bowel obstruction:

- History of perforated appendicitis or any other perforated abdominal viscus
- Crohn's Disease
- Severe gastrointestinal (GI) motility disorder such as severe gastroparesis
- Any history of actual, or suspected, bowel obstructions or small bowel surgery
- Any history of intraperitoneal adhesions

Conditions that predispose to gastric perforation:

- History of any previous bariatric, gastric or esophageal surgery
- History of previous laparoscopic band ligation
- History of anti-reflux surgery

GI bleeding or conditions that predispose to GI bleeding:

- Recent history of inflammatory conditions such as esophagitis, gastritis, gastric ulceration, or duodenal ulceration
- History of vascular lesions such as esophageal varices, gastric or duodenal varices, or intestinal telangiectasias
- Benign or malignant gastrointestinal tumors
- Inability to discontinue use of non-steroidal anti-inflammatory drugs (NSAIDs) or other gastric irritants during the device period
- Patients receiving anticoagulants
- Severe coagulopathy
- Hepatic insufficiency or cirrhosis
- Inability or unwillingness to take prescribed proton pump inhibitor medications in preparation for and/or during device residence

Other conditions:

- Serious or uncontrolled psychiatric illness
- Diagnosed bulimia, binge eating, compulsive overeating, or similar eating-related psychological disorders
- Alcoholism or drug addiction
- Pancreatitis
- Symptomatic congestive heart failure, cardiac arrhythmia, or unstable coronary artery disease
- Pre-existing significant respiratory disease such as chronic obstructive pulmonary disease (COPD), severe sleep apnea, or cystic fibrosis

- Cancer
- Known or suspected allergies to polyurethane
- Inability or unwillingness to take prescribed antiemetic medications in preparation for and/or during device residence
- Women who are pregnant or nursing
- Children younger than 18 years
- An existing gastric balloon that is currently in the stomach

Reference

1. Allurion Technologies. Allurion Gastric Balloon System, Instructions for Use. April 2021.
