# Supplementary material for: Impact of a Health Coach–Led, Text-Based Digital Behavior Change Intervention on Weight Loss and Psychological Well-Being in Patients Receiving a Procedureless Intragastric Balloon Program: Prospective Single-Arm Study
Source: JMIR Form Res. 2024 Jul 31;8:e54723. doi: 10.2196/54723 (PMC11325120; doi:10.2196/54723)
Supplement: Multimedia Appendix 2 [file formative_v8i1e54723_app2.pdf]

Multimedia Appendix 2: Attrition diagram.

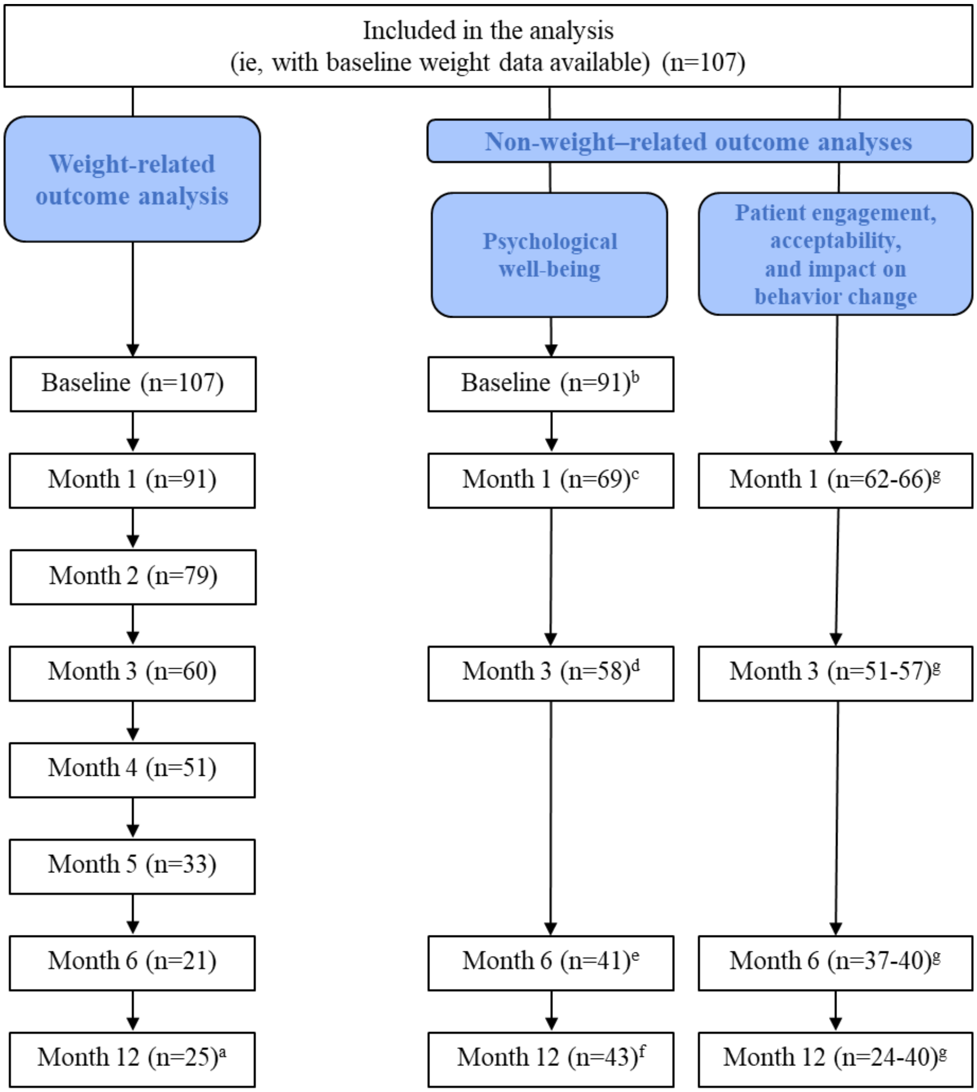

Only the time points in which data were collected and included in the analyses are shown in the diagram.

[a]At month 12, weight data were obtained via the connected scale (n=11 participants) and via self-reporting through the participant-reported satisfaction surveys (n=14 participants).

[b]Except GAD-7 (n=90), WEL-SF (n=83), and BBAQ (n=90).

[c]Except WEL-SF (n=68) and BBAQ (n=66).

[d]Except WEL-SF (n=57) and BBAQ (n=57).

[e]Except WEMWBS (n=42), GAD-7 (n=42), and BBAQ (n=40).

[f]Except IWQOL-Lite-CT overall and physical and psychosocial domains (n=42), LOCES-Brief (n=41); WEL-SF (n=40), and BBAQ (n=40).

[g]Range indicated due to space constraints; see Multimedia Appendix 3 for the number of participants with available responses for each item.

BBAQ, Barriers to Being Active Quiz; GAD-7, Generalized Anxiety Disorder 7-item Scale; IWQOL-Lite-CT, Impact of Weight on Quality of Life–Lite–Clinical Trials Version scale; LOCES-Brief, Loss of Control Over Eating Scale–Brief Version; WEL-SF, Weight Efficacy Lifestyle Questionnaire–Short Form.
