# Supplementary material for: Impact of a Health Coach–Led, Text-Based Digital Behavior Change Intervention on Weight Loss and Psychological Well-Being in Patients Receiving a Procedureless Intragastric Balloon Program: Prospective Single-Arm Study
Source: JMIR Form Res. 2024 Jul 31;8:e54723. doi: 10.2196/54723 (PMC11325120; doi:10.2196/54723)
Supplement: Multimedia Appendix 3 [file formative_v8i1e54723_app3.pdf]

Multimedia Appendix 3: Percentage of participants who scored positively each item of the participant-reported satisfaction surveys to measure engagement, acceptability of the intervention, and impact on behavior change.

	Proportion of participants rating the item with a positive score, % (n/N)[a]				
	Month 1	Month 3	Month 6	Average Months 1, 3, 6 (during DBCCI)	Month 12 (6 months after end of DBCCI)
**Items rated on a 5-point Likert scale**					
"I have found the health coaching useful/helpful"	82.81 (53/64)	80.39 (41/51)	82.50 (33/40)	81.90	NA
"I feel supported by my health coach towards meeting my weight goals" (Months 1, 3) and "My study health coaches helped me to meet my weight goals" (Month 6)	85.71 (54/63)	82.14 (46/56)	76.92 (30/39)	81.59	NA
"My health coach has helped me develop strategies to lose weight"	69.35 (43/62)	69.64 (39/56)	80.00 (32/40)	73.00	NA
"I feel confident that I can reach my goal weight and maintain it" (Months 1, 3 only)	67.69 (44/65)	66.07 (37/56)	NA	66.88	
"I feel confident about making changes to help me lose weight" (Months 1, 3) and "I feel confident about maintaining my weight loss" (Months 6, 12)	90.91 (60/66)	91.23 (52/57)	57.50 (23/40)	79.88	45.00 (18/40)
"I have been able to put the information and actions from the articles (weight loss actions) into practice"	76.56 (49/64)	62.96 (34/54)	56.76 (21/37)	65.43	NA
"I have made a plan to change my eating habits"	92.42 (61/66)	87.72 (50/57)	85.00 (34/40)	88.38	77.50 (31/40)
"I have made a plan to change my physical activity habits"	83.33 (55/66)	81.82 (45/55)	82.50 (33/40)	82.55	65.00 (26/40)
"I have made a plan to change my sleep habits"	51.61 (32/62)	34.62 (18/52)	42.11 (16/38)	42.78	43.59 (17/39)
"I am aware of some of the barriers to me reaching my weight goals"	89.39 (59/66)	89.47 (51/57)	74.36 (29/39)	84.41	55.00 (22/40)
"My health coaching was personalized to my needs"	71.43 (45/63)	73.21 (41/56)	53.85 (21/39)	66.16	NA
"I have found the articles (weight loss actions) sent by the health coaches useful/helpful"	78.12 (50/64)	68.52 (37/54)	66.67 (26/39)	71.10	NA

**Items rated on an 11-point Likert scale**					
"How satisfied have you been tracking your weight, using the connected scales, on a scale from 0 (not at all satisfied) to 10 (completely satisfied)?"	89.39 (59/66)	84.21 (48/57)	82.50 (33/40)	85.37	95.83 (23/24)

[a]A positive score was defined as a score ≥3 (ie, "slightly agree" or "strongly agree") for items rated on a 5-point Likert scale (0-4), or score ≥5 for items rated on an 11-point Likert scale (0-10; see Methods). DBCCI, digital behavior change coaching intervention; n, number of participants who gave a positive score; N, total number of participants with available responses; NA, not assessed.
